# Supplementary figures and images for: Glyoxal fixation facilitates transcriptome analysis after antigen staining and cell sorting by flow cytometry
Source: PLoS One. 2021 Jan 22;16(1):e0240769. doi: 10.1371/journal.pone.0240769 (PMC7822327; doi:10.1371/journal.pone.0240769)

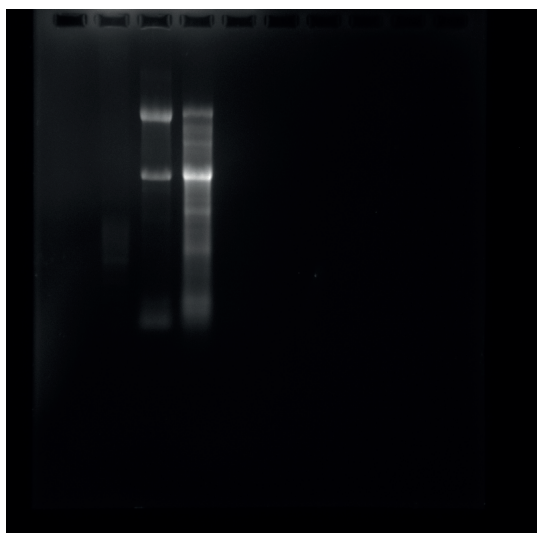

Fig. 2A

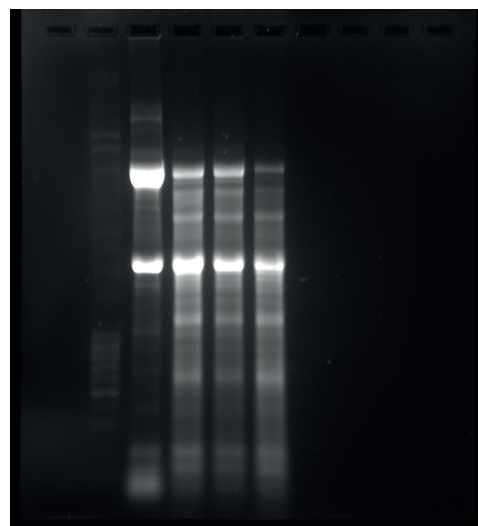

Fig. 2B (only lanes 2-4)

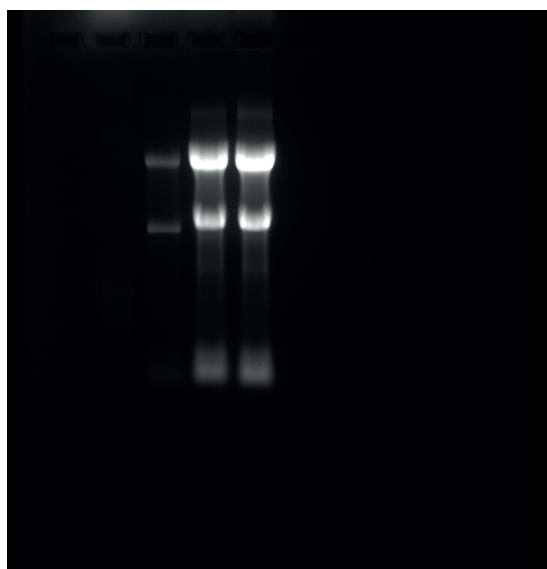

Fig. 2C

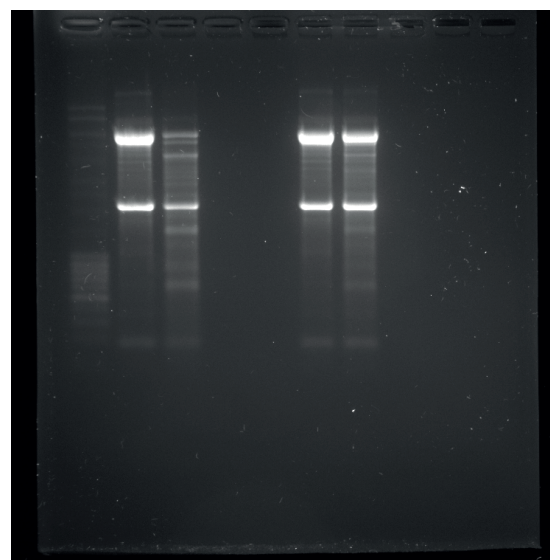

Fig 2D. (only lanes 6-8)

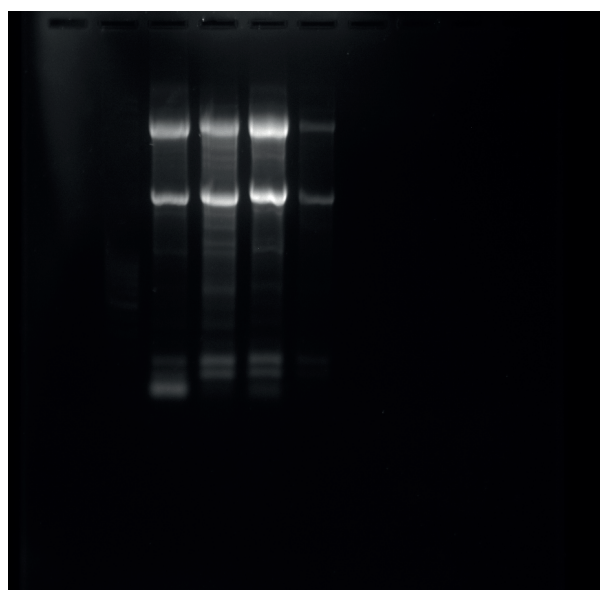

Fig. 2F

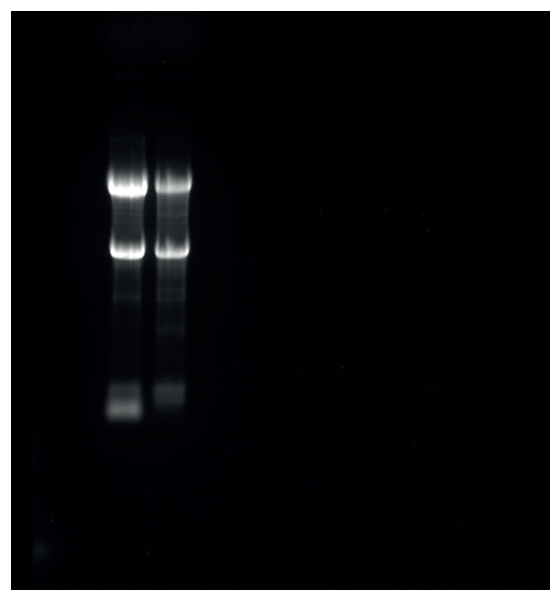

Fig 2G

Supplement: S1 Raw images — (PDF) [file pone.0240769.s001.pdf]
