# Supplementary material for: Glyoxal fixation facilitates transcriptome analysis after antigen staining and cell sorting by flow cytometry
Source: PLoS One. 2021 Jan 22;16(1):e0240769. doi: 10.1371/journal.pone.0240769 (PMC7822327; doi:10.1371/journal.pone.0240769)
Supplement: Attachment — Submitted filename: Rebuttal letter PLOS ONE.docx [file pone.0240769.s006.docx]

**Response to Reviewer #1**

The authors present here a convincing protocol for transcriptome analysis from cells, performed after fixation and antigen staining. The main point of the manuscript is to allow analyses that could not be performed using conventional formaldehyde fixation. This point is thoroughly presented by the present manuscript, but one aspect, the poor performance observed in formaldehyde-fixed samples, should be enhanced further. I suggest that the authors add the "formaldehyde" condition to their RT-PCR analysis in Figure 2E. This should make this point more convincing, and will therefore improve the manuscript.

We thank the reviewer for this suggestion and we have now added data in Figure 2E in order to demonstrate the poor performance of formaldehyde-fixed RNA in PCR amplification reactions. We had included this control in the original experiment but did not present the results, hence the data for the other two conditions remains the same and we have simply added the formaldehyde condition. Because the formaldehyde-fixed samples yielded almost no RNA as measured by a nanodrop, a volume of RNA sample equivalent to the control unfixed sample (from the same number of cells) was used for each RT-PCR reaction. The remarkable reduction in signals from formaldehyde-fixed samples compared to untreated and glyoxal-fixed samples is evident from the data. We have added text to include this information and corrected the figure legend for clarity.

**Response to Reviewer #2**

1) P. 4: “and library yields obtained were no more variable than we would normally expect between samples”. What is the expected and observed variability?

We find that yields with these RNAseq library preparation kits involving poly(A)+ enrichment are rather variable (over a 10-fold range or so), probably because they involve so many processing and purification steps. Furthermore yields vary substantially with cell type, user and batch of reagents. We would therefore be hesitant to define an expected range. However, we take the reviewer’s point that our phrasing suggests we have such a range, so we have re-phrased to simply quote the observed variability:

“library yields per cell type varied by <8-fold (3 amplification cycles)”

2) P. 4: “identified 2 and 61 differentially expressed genes”. If not already done please report the genes in a supplementary table. If table exists please cite it.

We have added this information in supplementary Table S1 and cited in the text.

3) P. 5: “An equivalent experiment performed with COLO205 cells will be described elsewhere.” I would recommend to either show the data or delete this statement.

We thank the reviewer for this recommendation. We included this statement as a manuscript describing this data is in preparation and we hoped to be able to cite the preprint. However, the preprint will not now be submitted in time and therefore we have deleted this statement

4) Glyoxal is reacting with RNA bases. Could the authors please comment on whether this creates mutations that may affect down-stream read-mapping or SNP calling?

This is a good point. As far as is known, the guanine-glyoxal adduct should revert to guanine; certainly the long history of using glyoxal as a denaturant in northern blotting means there cannot be a major level of mutation as that would impair hybridisation. We have looked at the mapping efficiencies in our various libraries; they are slightly reduced in the glyoxal fixed samples, but we think this is more likely due to the slight decrease in RNA quality caused by staining (visible in Fig. 2A and noted earlier in the manuscript). Nonetheless, we have only validated this method for calling RNA levels and would be wary of applying it for SNP calling without further validation. We have therefore added the following to the manuscript:

“Residual adducts would be expected to decrease the fidelity of reverse transcription, and resulting mutations to impair mapping. Indeed mapping efficiencies were slightly decreased in libraries prepared from fixed and stained samples, but the effect was small (unique mapped reads constituted 81±2% reads in libraries from unprocessed samples versus 75±5% in libraries made from fixed and stained samples). We suggest that this reduction stems from the slight decrease in RNA quality in stained samples, but further validation would be required before confidently calling SNPs from glyoxal-fixed RNA.”
