# Supplementary material for: Glyoxal fixation facilitates transcriptome analysis after antigen staining and cell sorting by flow cytometry
Source: PLoS One. 2021 Jan 22;16(1):e0240769. doi: 10.1371/journal.pone.0240769 (PMC7822327; doi:10.1371/journal.pone.0240769)
Supplement: Peer review history

# Peer review: Decision Letter 0

**DOI:** 10.1371/journal.pone.0240769.r001

**Authors:** Juan Mata

27 Oct 2020

PONE-D-20-30764

Glyoxal fixation facilitates transcriptome analysis after antigen staining and cell sorting by flow cytometry

PLOS ONE

Dear Jon,

Thank you for submitting your manuscript to PLOS ONE. After careful consideration, we feel that it has merit but does not fully meet PLOS ONE’s publication criteria as it currently stands. Therefore, we invite you to submit a revised version of the manuscript that addresses the very small points raised by the reviewers.

Please submit your revised manuscript by Dec 11 2020 11:59PM. If you will need more time than this to complete your revisions, please reply to this message or contact the journal office at plosone@plos.org. When you're ready to submit your revision, log on to https://www.editorialmanager.com/pone/ and select the 'Submissions Needing Revision' folder to locate your manuscript file.

Please include the following items when submitting your revised manuscript:

- A rebuttal letter that responds to each point raised by the academic editor and reviewer(s). You should upload this letter as a separate file labeled 'Response to Reviewers'.
- A marked-up copy of your manuscript that highlights changes made to the original version. You should upload this as a separate file labeled 'Revised Manuscript with Track Changes'.
- An unmarked version of your revised paper without tracked changes. You should upload this as a separate file labeled 'Manuscript'.

If you would like to make changes to your financial disclosure, please include your updated statement in your cover letter. Guidelines for resubmitting your figure files are available below the reviewer comments at the end of this letter.

If applicable, we recommend that you deposit your laboratory protocols in protocols.io to enhance the reproducibility of your results. Protocols.io assigns your protocol its own identifier (DOI) so that it can be cited independently in the future. For instructions see: http://journals.plos.org/plosone/s/submission-guidelines#loc-laboratory-protocols

We look forward to receiving your revised manuscript.

Kind regards,

Juan Mata

Academic Editor

PLOS ONE

Journal Requirements:

When submitting your revision, we need you to address these additional requirements.

1. Please ensure that your manuscript meets PLOS ONE's style requirements, including those for file naming. The PLOS ONE style templates can be found at

https://journals.plos.org/plosone/s/file?id=wjVg/PLOSOne_formatting_sample_main_body.pdf and

https://journals.plos.org/plosone/s/file?id=ba62/PLOSOne_formatting_sample_title_authors_affiliations.pdf

2. Please include captions for your Supporting Information files at the end of your manuscript, and update any in-text citations to match accordingly. Please see our Supporting Information guidelines for more information: http://journals.plos.org/plosone/s/supporting-information.

[Note: HTML markup is below. Please do not edit.]

Reviewers' comments:

Reviewer's Responses to Questions

**Comments to the Author**

1. Is the manuscript technically sound, and do the data support the conclusions?

The manuscript must describe a technically sound piece of scientific research with data that supports the conclusions. Experiments must have been conducted rigorously, with appropriate controls, replication, and sample sizes. The conclusions must be drawn appropriately based on the data presented. 

Reviewer #1: Yes

Reviewer #2: Yes

**********

2. Has the statistical analysis been performed appropriately and rigorously? 

Reviewer #1: Yes

Reviewer #2: Yes

**********

3. Have the authors made all data underlying the findings in their manuscript fully available?

The PLOS Data policy requires authors to make all data underlying the findings described in their manuscript fully available without restriction, with rare exception (please refer to the Data Availability Statement in the manuscript PDF file). The data should be provided as part of the manuscript or its supporting information, or deposited to a public repository. For example, in addition to summary statistics, the data points behind means, medians and variance measures should be available. If there are restrictions on publicly sharing data—e.g. participant privacy or use of data from a third party—those must be specified.

Reviewer #1: Yes

Reviewer #2: Yes

**********

4. Is the manuscript presented in an intelligible fashion and written in standard English?

PLOS ONE does not copyedit accepted manuscripts, so the language in submitted articles must be clear, correct, and unambiguous. Any typographical or grammatical errors should be corrected at revision, so please note any specific errors here.

Reviewer #1: Yes

Reviewer #2: Yes

**********

5. Review Comments to the Author

Please use the space provided to explain your answers to the questions above. You may also include additional comments for the author, including concerns about dual publication, research ethics, or publication ethics. (Please upload your review as an attachment if it exceeds 20,000 characters)

Reviewer #1: The authors present here a convincing protocol for transcriptome analysis from cells, performed after fixation and antigen staining.

The main point of the manuscript is to allow analyses that could not be performed using conventional formaldehyde fixation. This point is thoroughly presented by the present manuscript, but one aspect, the poor performance observed in formaldehyde-fixed samples, should be enhanced further. I suggest that the authors add the "formaldehyde" condition to their RT-PCR analysis in Figure 2E. This should make this point more convincing, and will therefore improve the manuscript.

Reviewer #2: This manuscript reports a detailed analysis for the use of the fixative Glyoxal for RNA studies. The authors provide first a thorough assessment of the impact of Glyoxal on RNA integrity alongside other fixative agents (Fig 2). They then use their protocol to performed RNA sequencing on FACS sorted cells stained for a cell-cycle marker (Fig 3). This demonstrates that glyoxal introduced only minimal, if any, biases to the transcriptome of treated cells while leading to good quality data.

This is a nice and thorough report that will be of very useful to the community. I particularly thank the authors for providing a detailed step by step protocol on their website. I only have a handful of minor comments which are listed below.

1) P. 4: “and library yields obtained were no more variable than we would normally expect between samples”. What is the expected and observed variability?

2) P. 4: “identified 2 and 61 differentially expressed genes”. If not already done please report the genes in a supplementary table. If table exists please cite it.

3) P. 5: “An equivalent experiment performed with COLO205 cells will be described elsewhere.”. I would recommend to either show the data or delete this statement.

4) Glyoxal is reacting with RNA bases. Could the authors please comment on whether this creates mutations that may affect down-stream read-mapping or SNP calling?

**********

6. PLOS authors have the option to publish the peer review history of their article (what does this mean?). If published, this will include your full peer review and any attached files.

If you choose “no”, your identity will remain anonymous but your review may still be made public.

**Do you want your identity to be public for this peer review?** For information about this choice, including consent withdrawal, please see our Privacy Policy.

Reviewer #1: **Yes: **Silvio Rizzoli

Reviewer #2: No

[NOTE: If reviewer comments were submitted as an attachment file, they will be attached to this email and accessible via the submission site. Please log into your account, locate the manuscript record, and check for the action link "View Attachments". If this link does not appear, there are no attachment files.]

While revising your submission, please upload your figure files to the Preflight Analysis and Conversion Engine (PACE) digital diagnostic tool, https://pacev2.apexcovantage.com/. PACE helps ensure that figures meet PLOS requirements. To use PACE, you must first register as a user. Registration is free. Then, login and navigate to the UPLOAD tab, where you will find detailed instructions on how to use the tool. If you encounter any issues or have any questions when using PACE, please email PLOS at figures@plos.org. Please note that Supporting Information files do not need this step.

# Author comment: Author response to Decision Letter 0

**DOI:** 10.1371/journal.pone.0240769.r002

10 Dec 2020

Response to Reviewer #1 

The authors present here a convincing protocol for transcriptome analysis from cells, performed after fixation and antigen staining. The main point of the manuscript is to allow analyses that could not be performed using conventional formaldehyde fixation. This point is thoroughly presented by the present manuscript, but one aspect, the poor performance observed in formaldehyde-fixed samples, should be enhanced further. I suggest that the authors add the "formaldehyde" condition to their RT-PCR analysis in Figure 2E. This should make this point more convincing, and will therefore improve the manuscript.

We thank the reviewer for this suggestion and we have now added data in Figure 2E in order to demonstrate the poor performance of formaldehyde-fixed RNA in PCR amplification reactions. We had included this control in the original experiment but did not present the results, hence the data for the other two conditions remains the same and we have simply added the formaldehyde condition. Because the formaldehyde-fixed samples yielded almost no RNA as measured by a nanodrop, a volume of RNA sample equivalent to the control unfixed sample (from the same number of cells) was used for each RT-PCR reaction. The remarkable reduction in signals from formaldehyde-fixed samples compared to untreated and glyoxal-fixed samples is evident from the data. We have added text to include this information and corrected the figure legend for clarity.

Response to Reviewer #2 

1) P. 4: “and library yields obtained were no more variable than we would normally expect between samples”. What is the expected and observed variability?

We find that yields with these RNAseq library preparation kits involving poly(A)+ enrichment are rather variable (over a 10-fold range or so), probably because they involve so many processing and purification steps. Furthermore yields vary substantially with cell type, user and batch of reagents. We would therefore be hesitant to define an expected range. However, we take the reviewer’s point that our phrasing suggests we have such a range, so we have re-phrased to simply quote the observed variability:

“library yields per cell type varied by <8-fold (3 amplification cycles)”

2) P. 4: “identified 2 and 61 differentially expressed genes”. If not already done please report the genes in a supplementary table. If table exists please cite it.

We have added this information in supplementary Table S1 and cited in the text. 

3) P. 5: “An equivalent experiment performed with COLO205 cells will be described elsewhere.” I would recommend to either show the data or delete this statement.

We thank the reviewer for this recommendation. We included this statement as a manuscript describing this data is in preparation and we hoped to be able to cite the preprint. However, the preprint will not now be submitted in time and therefore we have deleted this statement 

4) Glyoxal is reacting with RNA bases. Could the authors please comment on whether this creates mutations that may affect down-stream read-mapping or SNP calling?

This is a good point. As far as is known, the guanine-glyoxal adduct should revert to guanine; certainly the long history of using glyoxal as a denaturant in northern blotting means there cannot be a major level of mutation as that would impair hybridisation. We have looked at the mapping efficiencies in our various libraries; they are slightly reduced in the glyoxal fixed samples, but we think this is more likely due to the slight decrease in RNA quality caused by staining (visible in Fig. 2A and noted earlier in the manuscript). Nonetheless, we have only validated this method for calling RNA levels and would be wary of applying it for SNP calling without further validation. We have therefore added the following to the manuscript:

“Residual adducts would be expected to decrease the fidelity of reverse transcription, and resulting mutations to impair mapping. Indeed mapping efficiencies were slightly decreased in libraries prepared from fixed and stained samples, but the effect was small (unique mapped reads constituted 81±2% reads in libraries from unprocessed samples versus 75±5% in libraries made from fixed and stained samples). We suggest that this reduction stems from the slight decrease in RNA quality in stained samples, but further validation would be required before confidently calling SNPs from glyoxal-fixed RNA.”

Attachment Submitted filename: Rebuttal letter PLOS ONE.docx Click here for additional data file.

# Editor report: Decision Letter 1

**DOI:** 10.1371/journal.pone.0240769.r003

**Authors:** Juan Mata

5 Jan 2021

Glyoxal fixation facilitates transcriptome analysis after antigen staining and cell sorting by flow cytometry

PONE-D-20-30764R1

Dear Jon,

We’re pleased to inform you that your manuscript has been judged scientifically suitable for publication and will be formally accepted for publication once it meets all outstanding technical requirements.

Within one week, you’ll receive an e-mail detailing the required amendments. When these have been addressed, you’ll receive a formal acceptance letter and your manuscript will be scheduled for publication.

An invoice for payment will follow shortly after the formal acceptance. To ensure an efficient process, please log into Editorial Manager at http://www.editorialmanager.com/pone/, click the 'Update My Information' link at the top of the page, and double check that your user information is up-to-date. If you have any billing related questions, please contact our Author Billing department directly at authorbilling@plos.org.

If your institution or institutions have a press office, please notify them about your upcoming paper to help maximize its impact. If they’ll be preparing press materials, please inform our press team as soon as possible -- no later than 48 hours after receiving the formal acceptance. Your manuscript will remain under strict press embargo until 2 pm Eastern Time on the date of publication. For more information, please contact onepress@plos.org.

Kind regards,

Juan Mata, Ph.D.

Academic Editor

PLOS ONE

Additional Editor Comments (optional):

Reviewers' comments:

# Editor report: Acceptance letter

**DOI:** 10.1371/journal.pone.0240769.r004

**Authors:** Juan Mata

11 Jan 2021

PONE-D-20-30764R1 

Glyoxal fixation facilitates transcriptome analysis after antigen staining and cell sorting by flow cytometry 

Dear Dr. Houseley:

I'm pleased to inform you that your manuscript has been deemed suitable for publication in PLOS ONE. Congratulations! Your manuscript is now with our production department. 

If your institution or institutions have a press office, please let them know about your upcoming paper now to help maximize its impact. If they'll be preparing press materials, please inform our press team within the next 48 hours. Your manuscript will remain under strict press embargo until 2 pm Eastern Time on the date of publication. For more information please contact onepress@plos.org.

If we can help with anything else, please email us at plosone@plos.org. 

Thank you for submitting your work to PLOS ONE and supporting open access. 

Kind regards, 

PLOS ONE Editorial Office Staff

on behalf of

Dr. Juan Mata 

Academic Editor

PLOS ONE
